# Experimental Evaluation on Depth Control Using Improved Model Predictive Control for Autonomous Underwater Vehicle (AUVs)

**DOI:** 10.3390/s18072321

**Published:** 2018-07-17

**Authors:** Feng Yao, Chao Yang, Xing Liu, Mingjun Zhang

**Affiliations:** College of Mechanical and Electrical Engineering, Harbin Engineering University, Nangang District, Harbin 150001, China; a0232072590@126.com (C.Y.); liuxing0724@hrbeu.edu.cn (X.L.); zhangmingjun@hrbeu.edu.cn (M.Z.)

**Keywords:** AUV, model predictive control, control increment vector weighting matrix, trajectory tracking

## Abstract

Due to the growing interest using model predictive control (MPC), there are more and more researches about the applications of MPC on autonomous underwater vehicle (AUV), and these researches are mainly focused on simulation and simple application of MPC on AUV. This paper focuses on the improvement of MPC based on the state space model of an AUV. Unlike the previous approaches using a fixed weighting matrix, in this paper, a coefficient, varied with the error, is introduced to adjust the control increment vector weighting matrix to reduce the settling time. Then, an analysis on the effect of the adjustment to the stability is given. In addition, there is always a lag between the AUV real trajectory and the desired trajectory when the AUV tracks a continuous trajectory. To solve this problem, a simple re-planning of the desired trajectory is developed. Specifically, the point certain steps ahead from current time on the desired trajectory is treated as the current desired point and input to the controller. Finally, experimental results for depth control are given to demonstrate the feasibility and effectiveness of the improved MPC. Experimental results show that the method of real-time adjusting control increment weighting matrix can reduce settling time by about 2 s when tracking step trajectory of 1 m, and the simple re-planning of the desired trajectory method can reduce the average of absolute error by about 15% and standard deviation of error by about 17%.

## 1. Introduction

In the complex deep sea, autonomous underwater vehicle (AUVs) are the unique solution for various missions, including seabed topographic survey, environmental monitoring, resource exploration, target search [1,2], and with underwater wireless sensor networks, AUV also can be applied into researches on abyssal habitats and aquaculture monitoring [3,4]. Operating in a complex marine environment, AUVs need to execute underwater missions autonomously, such as detection, cruise, and operation. Therefore, motion control is a significant important part for AUVs to complete their tasks [5,6].

A current topic in AUVs motion control is to improve the control accuracy, convergence speed of the controller, and the adaptability of AUVs. Nowadays, many control approaches have been proposed for motion control of AUVs, such as sliding mode control [7,8], neural network control [9,10], fuzzy control [11,12], and so on, which have been successfully used to improve the accuracy and convergence speed for some cases. In the sliding mode controller, the control output always has serious chattering phenomenon. Hence, the reduction of chattering phenomenon is an important issue in sliding mode design [13,14,15]. In fuzzy based controller, the fuzzy rules rely on the designer’s experience and these rules also need to be adjusted on-line. In the neural network based controller, the weighing of network need to be trained off-line according to the historical experiment data. Furthermore, these three controllers do not have good results to address actuator constraints. In these control approaches, control constraints did not considered in controller design. Sometimes in order to satisfy the control constraints, some control performance would be sacrificed [16]. The most attractive feature of model predictive control (MPC) lies in its ability of handling constraints explicitly, which comes from the prediction for future behavior [17]. With the receding horizon control principle, MPC can effectively deal with the complex disturbances from marine environment, nonlinearity of AUVs’ system, model mismatch, and multi constrains. All of these approaches encounter a problem that there is always a lag between the real trajectory and the desired trajectory. Many scholars focus on reducing the lag between the actual trajectory and the desired trajectory by optimizing control approaches to improve the control accuracy [18,19,20].

In [21], a novel MPC scheme using reduced dynamical model is proposed to investigate the motion control problem of AUV, and the simulation result demonstrates the validity of the scheme in three-dimensional space. MPC based on the state space model is applied to improve the control performance of AUV or remote operated vehicle (ROV) in [22,23], and simulation proves the effectiveness of MPC in heading and depth control. Reference [24] gave the simulation results based on the MPC-based controller and confirmed the feasibility of the MPC-based controller in comparison with the results that are based on PID controller. Different from these simulation evaluations, in [16,25,26], MPC based on the state space model is applied in the control experiment of AUV or ROV, and the experimental results provide clear evidence to show that the MPC algorithm can be successfully implemented on AUVs.

This paper focuses on the improvement of MPC by varying control increment vector weighting matrix, according to the error and simply re-planning the desired trajectory to reduce the lag between the AUV real trajectory and the desired trajectory. Furthermore, the improved MPC is implemented on UVIC-I AUV [27] in a pool for experimental tests to validate the effectiveness of the proposed MPC methods.

This paper is organized, as follows. Section 2 describes MPC by using an AUV’s state-space model, and here only one freedom of degree (Heave) is considered. Research on the approach of adjusting the control increment weighting matrix vector is presented in Section 3. Section 4 describes the approach of simply re-planning the desired trajectory. In Section 5, the effectiveness of the proposed approaches is verified by experiment results. Finally, we make a brief conclusion of the paper in Section 6.

## 2. Problem Formulation

In this section, the discrete-time state space model of the AUVs’ depth is established on the base of dynamic equation. Then the MPC controller is designed, followed by the analysis about the weakness of the control law.

MPC is initially applied in process control of refining, chemical, and electric power. With the development of MPC, in recent years, it is applied in the areas of aerospace, advanced manufacturing, medicine, and so on, such as flight control [28], satellite attitude control [29], blood glucose control [30], etc. The current research emphasis of MPC is to solve online constrained optimization and an investigation of new ideas and methods to overcome the limitations of MPC [17]. This paper is addressed on the experimental evaluation of the improved MPC on AUV.

### 2.1. Discrete-Time State Space Model of AUVs’ Depth

According to reference [31], the dynamic equation of AUVs’ depth can be described as:

(1) $$m_{z}\ddot{z}+\left(C_{z}\left(v\right)+D_{z}\left(v\right)\right)\dot{z}=\tau_{z}-g_{z}\left(\eta \right)$$

where *m_z_* is the inertia matrix in depth, *z* is depth of AUV, ***v*** is the linear and angular velocity vector with coordinates in the body-fixed frame, ***η*** is the position and orientation vector with coordinates in the earth-fixed frame, *C_z_*(***v***) is the Coriolis and centripetal matrix in depth, *D_z_*(***v***) is the damping matrix in depth, *τ_z_* is the control input in depth, and *g_z_*(***η***) is the gravitational force in depth.

To simplify the formulation, let *f* = *C_z_*(***v***) + *D_z_*(***v***), *u* = *τ_z_* − *g_z_*(***η***), Equation (1) can be expressed as the following compact form:

(2) $$m_{z}\ddot{z}+f\dot{z}=u$$

where *z* is the depth and *m_z_* and *f* are obtained by the model of AUVs’ depth recognized by experiments.

Equation (2) can be written in a continuous-time state space form:

(3) $$\begin{array}{l} \dot{x}=\left[\begin{array}{l} \dot{z}\\ \ddot{z} \end{array}\right]=A_{c}\left[\begin{array}{c} z\\ \dot{z} \end{array}\right]+B_{cu}u=\left[\begin{array}{cc} 0 & 1\\ 0 & -m_{z}{}^{-1}f \end{array}\right]\left[\begin{array}{c} z\\ \dot{z} \end{array}\right]+\left[\begin{array}{c} 0\\ m_{z}{}^{-1} \end{array}\right]u\\ y=C_{c}\left[\begin{array}{c} z\\ \dot{z} \end{array}\right]=\left[\begin{array}{cc} 1 & 0 \end{array}\right]\left[\begin{array}{c} z\\ \dot{z} \end{array}\right] \end{array}\;$$

where ***x***, *u*, *y* are the continuous-time state, input and output vectors, respectively, ***A****_c_* is state matrix, ***B****_cu_* is the input-to-state matrix, and ***C****_c_* is the state-to-output matrix.

The discrete-time state space form of Equation (3) is written as Equation (4):

(4) $$\begin{array}{l} x\left(k+1\right)=\left[\begin{array}{l} z\left(k+1\right)\\ v_{z}\left(k+1\right) \end{array}\right]=A\left[\begin{array}{c} z\left(k\right)\\ v_{z}\left(k\right) \end{array}\right]+Bu\left(k\right)\\ y\left(k\right)=C\left[\begin{array}{c} z\left(k\right)\\ v_{z}\left(k\right) \end{array}\right] \end{array}$$

where

$$A=e^{A_{c}T}=\left[\begin{array}{cc} 1 & \frac{e^{-m_{z}{}^{-1}f\left(k\right)T_{c}}-1}{-m_{z}{}^{-1}f\left(k\right)}\\ 0 & e^{-m_{z}{}^{-1}f\left(k\right)T_{c}} \end{array}\right]$$

$$B=\int_{0}^{T}e^{A_{c}\tau }B_{cu}d\tau =\left[\begin{array}{c} \frac{e^{-m_{z}{}^{-1}f\left(k\right)T_{c}}+m_{z}{}^{-1}f\left(k\right)T_{c}-1}{m_{z}{}^{-1}f{\left(k\right)}^{2}}\\ \frac{e^{-m_{z}{}^{-1}f\left(k\right)T_{c}}-1}{-f\left(k\right)} \end{array}\right]$$

$$C=C_{c}$$

*v_z_*(*k*) is the linear velocity with coordinates in the body-fixed frame in discrete-time domain, *k* is the time in discrete-time domain, ***x***(*k*), *u*(*k*), *y*(*k*) are the discrete-time state, input and output vectors, respectively, and *T*_c_ is the control cycle.

The MPC algorithm that was used in this work embeds an integrator into the model to ensure zero steady-state errors for desired point tracking [32,33], and then the discrete-time state space model used in design can be expressed as Equation (5):

(5) $$\begin{array}{l} \Delta x\left(k+1\right)=A\Delta x\left(k\right)+B\Delta u\left(k\right)\\ y\left(k\right)=C\Delta x\left(k\right)+y\left(k-1\right) \end{array}$$

where Δ***x***(*k*) = ***x***(*k*) − ***x***(*k* − 1) and Δ*u*(*k*) = *u*(*k*) − *u*(*k* − 1).

### 2.2. MPC Algorithm Control Law

According to [32,33], the cost function used in this paper is shown in Equation (6):

(6) $$J={\left\|\Gamma_{y}\left(Y_{p}\left(k+1\right)-R\left(k+1\right)\right)\right\|}^{2}+{\left\|\Gamma_{u}\Delta U\left(k\right)\right\|}^{2}$$

where ***Y****_p_*(*k* + 1) is the predicted output vector with a prediction horizon of *p* at sample time *k*, *p* is the prediction horizon, ***R***(*k* + 1) is the given reference vector at sample time *k* and ***R***(*k* + 1) = [*r*(*k* + 1)^T^ *r*(*k* + 2)^T^ *r*(*k* + 3)^T^ … *r*(*k* + *p*)^T^]^T^, T represents the transpose of the matrix. **Δ*U***(*k*) is the control increment vector at sample time *k* and **Δ*U***(*k*) = [Δ*u*(*k*)^T^ Δ*u*(*k* + 1)^T^ Δ*u*(*k* + 2)^T^ … Δ*u*(*k* + *m*)^T^]^T^, *m* is the control horizon. ***Γ****_y_* and ***Γ**_u_* are weight diagonal matrices for the predictive error vector and control increment vector, respectively, which are usually taken as constant matrices with compatible dimensions [16,22,32]. In this paper, according to [16,23,32], ***Γ****_y_* is an identity matrix, while the weight ***Γ**_u_* is adjusted based on the error, and more details will be given in the later.

Routine analysis gives the solution of the control increment vector as Equation (7):

(7) $$\Delta U\left(k\right)={\left(S_{u}^{T}\Gamma_{y}^{T}\Gamma_{y}S_{u}+\Gamma_{u}^{T}\Gamma_{u}\right)}^{-1}S_{u}^{T}\Gamma_{y}^{T}\Gamma_{y}E_{p}\left(k+1\right)={\left(S_{u}^{T}S_{u}+\Gamma_{u}^{T}\Gamma_{u}\right)}^{-1}S_{u}^{T}E_{p}\left(k+1\right)$$

where

$$S_{u}=\left[\begin{array}{ccccc} CB & 0 & 0 & \ldots & 0\\ \sum_{i=1}^{2}CA^{i-1}B & CB & 0 & \ldots & 0\\ \ldots & \ldots & \ldots & \ldots & 0\\ \sum_{i=1}^{m}CA^{i-1}B & \sum_{i=1}^{m-1}CA^{i-1}B & \ldots & \ldots & CB\\ \ldots & \ldots & \ldots & \ldots & \ldots \\ \sum_{i=1}^{p}CA^{i-1}B & \sum_{i=1}^{p-1}CA^{i-1}B & \ldots & \ldots & \sum_{i=1}^{p-m+1}CA^{i-1}B \end{array}\right]$$

$$E_{p}\left(k+1\right)=R\left(k+1\right)-S_{x}\Delta x\left(k\right)-I_{c}y\left(k\right)$$

$$S_{x}=\left[\begin{array}{c} CA\\ \sum_{i=1}^{2}CA^{i}\\ \vdots \\ \sum_{i=1}^{p}CA^{i} \end{array}\right];\;I_{c}=\left[\begin{array}{c} 1\\ 1\\ \vdots \\ 1 \end{array}\right]$$

Using receding horizon control, ∆*u*(*k*), the first sub-vector of **∆*U***(*k*), is computed by Equation (8).

(8) $$\Delta u\left(k\right)=\left[\begin{array}{cccc} 1 & 0 & \cdots & 0 \end{array}\right]{\left(S_{u}^{T}S_{u}+\Gamma_{u}^{T}\Gamma_{u}\right)}^{-1}S_{u}^{T}E_{p}\left(k+1|k\right)$$

Then the actual control vector applied to the plant is computed by Equation (9):

(9) u(k)=u(k−1)+Δu(k) 

where *u*(*k* − 1) is the past input vector.

As can be seen in Equation (8), in conventional MPC method, the weight matrix ***Γ**_u_* and the weight matrix ***Γ****_y_* are fixed during the control [33]. However, in the design of cost-function, three points are considered. Firstly, we want to reduce the term ||***Y****p*(*k* + 1)−***R***(*k* + 1)||^2^ as small as possible in the whole process. Secondly, the term ||Δ***U***(*k*)||^2^ in the transient stage should be paid more attention than that in the steady stage. Thirdly, in the transient stage, the attention that is given to the term ||Δ***U***(*k*)||^2^ should be varied. Therefore, the control objective of this paper is to adjust ***Γ**_u_*, according to the error on-line and to decrease the lag between the real trajectory and the desired trajectory.

## 3. MPC Method with a Real-Time Adjusting Control Increment Vector Weighting Matrix

This section is addressed on the approach of real-time adjustment of ***Γ**_u_* (i.e., the control increment vector weighting matrix), and then analysis on the effect of the adjustment for ***Γ**_u_* on the stability is presented.

MPC is a typical control algorithm, which is widely applied to many nonlinear systems. But, there are not many published researches about MPC based control for AUVs, e.g., [16,26]. In these above references, the weights (***Γ****_y_*, ***Γ**_u_*) of the terms ||***Y****_p_*(*k* + 1) − ***R***(*k* + 1)||^2^ and ||Δ***U***(*k*)||^2^ in the cost function are fixed. In this paper, we want to adjust the weights ratio between the term ||***Y****_p_*(*k* + 1) − ***R***(*k* + 1)||^2^ and the term ||Δ***U***(*k*)||^2^, according to the tracking error. In this paper, the weight (***Γ****_y_*) in the term ||***Y****_p_*(*k* + 1) − ***R***(*k* + 1)||^2^ is fixed identity matrix, while the weight (***Γ**_u_*) in the term ||Δ***U***(*k*)||^2^ is varied. Specifically, in the case of a large tracking error, a small value (less than 1) is determined on-line based on tracking error for the weight (***Γ**_u_*) to reduce the term ||***Y****_p_*(*k* + 1) − ***R***(*k* + 1)||^2^ as soon as possible, i.e., to reduce the settling time. When the tracking error is small, the weight (***Γ**_u_*) is set as an identity matrix. As discussed in experimental verification, the improved MPC method is an optional for some special AUV applications, which focus on settling time and tracking precision.

A large ***Γ**_u_* will result in that the control increment vector is too conservative during the settling time, leading to long settling time. If ***Γ**_u_* is small, AUVs will be very sensitive to the environment disturbances and small fluctuations from the sensor during the steady state, which means that the constraint of control increment vector helps to reduce disturbances and fluctuations. In this section, an approach of adjusting ***Γ**_u_* according to the error is proposed. A coefficient varying with the error is introduced to adjust ***Γ**_u_*, not only to ensure that the control increment vector is not conservative, improving the system’s dynamic response performance, but also to avoid bringing fluctuations into the steady state.

### 3.1. Adjusting **Γ_u_** According to the Error

In this paper, the MPC method with a real-time adjusting ***Γ**_u_* is realized by introducing a coefficient *α* to ***Γ**_u_*. ***Γ**_u_* can be expressed as Equation (10):

(10) $$\Gamma_{u}=\alpha \cdot I_{m\times m}$$

where ***I**_m×m_* is *m*-dimensional identity matrix and *α* is tuned online according to the error of the current time referring to certain rules.

**Figure 1:**
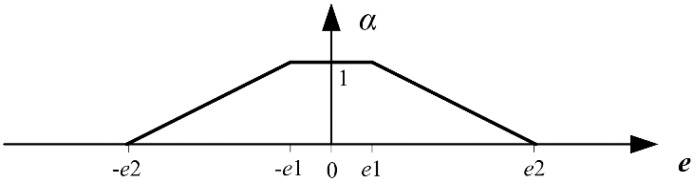
Relationship curve of *α* and *e* used in this paper.

According to the control precision requirements of AUV, two boundary values of depth error, *e*1 and *e*2, are determined, and *e*2 > *e*1 > 0. *e* denotes the current error of depth. When the error is large, i.e., ||*e*|| ≥ *e*2, in order to improve the response speed of the system, it is necessary to reduce the weight of control increment vector. In this case, *α* is set as 0. When the error is small, i.e., ||*e*|| ≤ *e*1, in order to avoid fluctuations that are caused by quick changes of input in the steady state, it needs to increase the weight of control increment vector. In this case *α* is set as 1. In order to obtain a smooth transition for *α* from *α* = 0 to *α* = 1, when *e*1 < ||*e*|| < *e*2, a linear function is used to determine the value of *α* shown as Figure 1, and the adjustment equation of *α* is shown as Equation (11).

(11) $$\left\{ \begin{array}{ll} \alpha =0 & \left(\left\|e\right\|\geq e2\right)\\ \alpha =\frac{e2-\left\|e\right\|}{e2-e1} & \left(e1<\left\|e\right\|<e2\right)\\ \alpha =1 & \left(\left\|e\right\|\leq e1\right) \end{array}\right.$$

### 3.2. Stability Analysis for Adjusting **Γ**_u_

To discuss the stability of the system after adjusting ***Γ**_u_*, substitute Equation (10) into Equation (8), and yield to Equation (12):

(12) $$\Delta u\left(k\right)=\left[\begin{array}{cccc} 1 & 0 & \cdots & 0 \end{array}\right]{\left(S_{u}^{T}S_{u}+\Gamma_{u}^{T}\Gamma_{u}\right)}^{-1}S_{u}^{T}E_{p}\left(k+1|k\right)=K_{mpc}E_{p}\left(k+1|k\right)$$

By using Equations (5) and (12), ∆***x***(*k* + 1) is expressed as Equation (13):

(13) $$\Delta x\left(k+1\right)=\left(A-BK_{mpc}\left(S_{x}+I_{c}C\right)\right)\Delta x\left(k\right)+BK_{mpc}R\left(k+1\right)-BK_{mpc}I_{c}Cx\left(k-1\right)$$

According to [34,35], if all the eigenvalues of the matrix ***A*** − ***BK****_mpc_*(***S****_x_* + ***I****_c_**C***) are in the unit circle in the complex plane, the system is asymptotically stable. ***A***, ***B***, ***C*** used in this paper are described in Section 5.2, determined according to the dynamic model of the experimental AUV.

**Figure 2:**
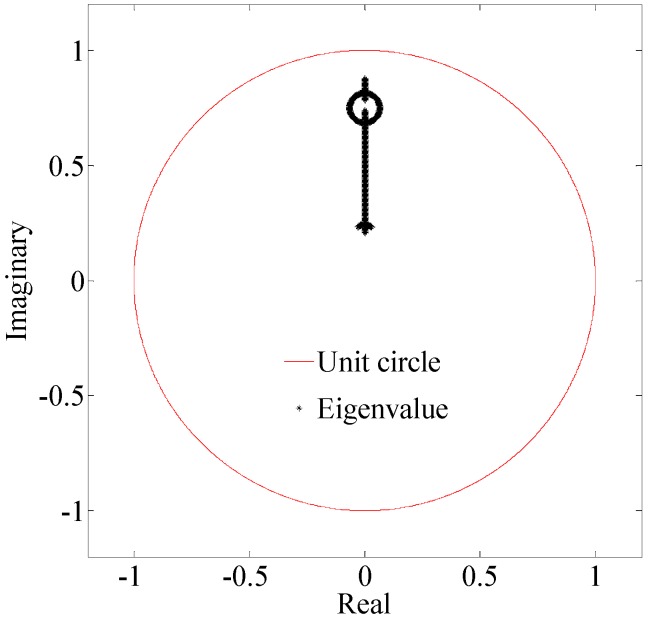
Relationship between the eigenvalues of ***A*** − ***BK****_mpc_*(***S****_x_* + ***I**_c_**C***) and unit circle in the complex plane.

Figure 2 shows the relationship between the eigenvalues of ***A*** − ***BK****_mpc_*(***S****_x_* + ***I****_c_**C***) and unit circle in the complex plane when *α* varies from 0 to 1. From Figure 2, it can be seen that all of the eigenvalues are in the unit circle. It indicates that the system with an adjusting ***Γ**_u_* is asymptotically stable.

## 4. Simply Re-Planning the Desired Trajectory to Reduce the Lag Component

In this section, the method of simply re-planning the desired trajectory is discussed to reduce the lag component, including the reason of re-choosing another desired point real-time and how to determine this point.

More analysis on the experimental data from the MPC method in reference [16,26] shows that: During tracking a trajectory, there always a time lag between the output trajectory and the desired trajectory. In this section, an approach of simply re-planning the desired trajectory is proposed to reduce the lag component. In this approach, firstly, choose *N* (i.e., the count of steps ahead in simply re-planning the desired trajectory method), according the experimental data, and then in subsequent control, set the desired point *N* steps ahead as the desired point for current time.

### 4.1. The Reason of Re-Choosing a Current Desired Point

**Figure 3:**
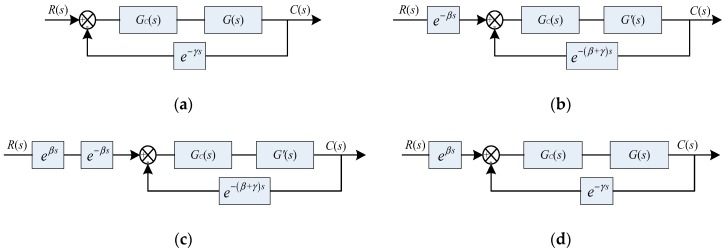
Evolution of autonomous underwater vehicle (AUVs) control system block diagram in this paper. (**a**) AUVs’ block diagram; (**b**) block diagram after separating the lag component and moving the summing junction past the *e*^−*βs*^ block; (**c**) block diagram after introducing *e*^−*βs*^ component between *R*(*s*) and the summing junction; and, (**d**) final block diagram after introducing *e*^−*βs*^ component.

To simplify analysis, in this paper, AUVs’ block diagram is simplified, as shown in Figure 3a, where *R*(*s*) and *C*(*s*) are the desired-trajectory and the output trajectory, respectively, as described in frequency domain. *G*(*s*) is the transfer function of AUVs and *G_C_*(*s*) is the transfer function of the control algorithm, and *e*^−*γs*^ is the lag component of sensor. Separating the lag component *e*^−*βs*^ (including the lag components of the thruster, the vehicle, the control circuit, etc.) block of AUVs and moving the summing junction past the *e*^−*βs*^ block, Figure 3a can be transformed into Figure 3b, where *G*′(*s*) is the transfer function of AUVs after removing the lag component and *e*^−*βs*^ is the transfer function of lag component.

It can be considered that the desired-trajectory has a lag component of *e*^−*βs*^ and the sensor has lag component of *e*^−(*β*+*γ*)*s*^, but AUVs do not have any lag component. In this paper, we do not solve the problem of sensor’s lag. The lag component of desired trajectory can be eliminated by introducing *e^βs^* component between *R*(*s*) and the summing junction, as shown in Figure 3c, which can be implemented by re-planning of the desired trajectory, i.e., treating the point certain steps ahead on the desired trajectory as the present desired point and input of controller in the discrete-time domain. This paper uses parameter *N* to represent the certain steps. At time *t*, input is calculated by the error between output at time *t* and the desired point at time *t* + *N* × *T*_c_, i.e., the desired point at time *t* is the point at time *t* + *N* × *T*_c_ on the desired trajectory. *T*_c_ is control interval and *N* is determined by the experimental data, as is shown in Section 4.2.

Moving back the *e*^−*βs*^ block past the summing junction, the equivalent block diagram of Figure 3c is obtained, as shown in Figure 3d, and the block diagram shown in Figure 3d is used for AUV trajectory tracking control.

The transfer function of the original block diagram without any process shown in Figure 3a is:

(14) $$\frac{C\left(s\right)}{R\left(s\right)}=\frac{G_{C}\left(s\right)G\left(s\right)}{1+G_{C}\left(s\right)G\left(s\right)e^{-\gamma s}}$$

The transfer function after simply re-planning the desired trajectory shown in Figure 3d is:

(15) $$\frac{C\left(s\right)}{R\left(s\right)}=\frac{e^{\beta s}G_{C}\left(s\right)G\left(s\right)}{1+G_{C}\left(s\right)G\left(s\right)}=\frac{\left(1+\beta s+\frac{\beta^{2}s^{2}}{2!}+\frac{\beta^{3}s^{3}}{3!}+\cdots \right)G_{C}\left(s\right)G\left(s\right)}{1+G_{C}\left(s\right)G\left(s\right)e^{-\gamma s}}$$

In Equation (15), the introduction of *e^βs^* is equivalent to the introduction of differential and high-order differential components, i.e., adding predictive function to the control system. It indicates that the introduction of *e^βs^* can improve the response speed and the trajectory tracking performance of the system.

When comparing Equations (14) and (15), it can be seen that they have same poles, which means that the method of simply re-planning the desired trajectory proposed in this paper has no effect on the stability.

### 4.2. How to Determine the Parameter N

**Figure 4:**
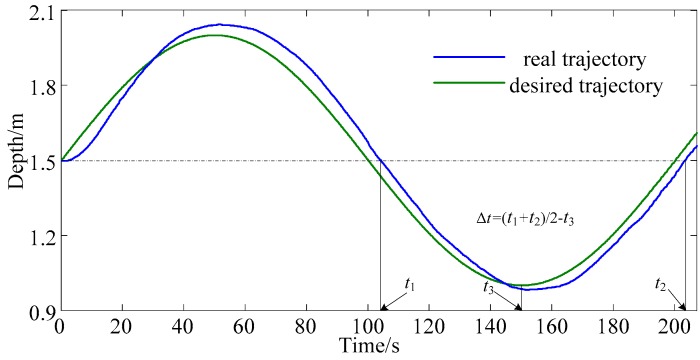
Experimental result curve on depth control with no improvement.

Parameter *N* is calculated by *N* = Δ*t*/*T*_c_, where Δ*t* is the lag time between the desired trajectory and the experimental output curve, shown in Figure 4, Δ*t* = (*t*_1_ + *t*_2_)/2 − *t*_3_. In Figure 4, *t*_1_ and *t*_2_ is the time when the depth of the AUV reaches the equilibrium position of desired sinusoidal trajectory, and *t*_3_ is the peak time of desired sinusoidal trajectory.

**Figure 5:**
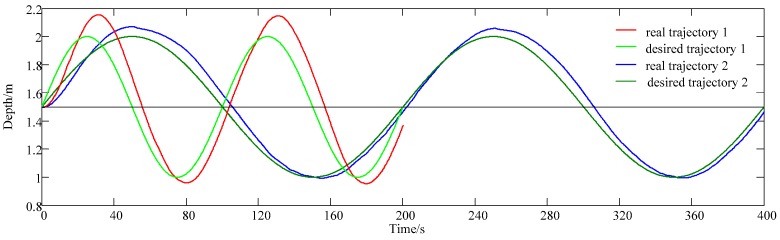
Experimental result of tracking the sinusoidal trajectory with periods of 100 s and 200 s. The period of trajectory 1 is 100 s and the period of trajectory 2 is 200 s.

Experiments are conducted to obtain Δ*t* and *N*. First, the AUV is fixed at 1.5 m and then tracks a sinusoidal trajectory. Δ*t* is measured by the experimental data, and *N* is calculated by *N* = Δ*t*/*T*_c_. It is found from experiments that when tracking different frequency sinusoidal trajectories, the difference in the lag time is not large. Take tracking the sinusoidal trajectory with periods of 100 s and 200 s as an example, as shown in Figure 5, where the period of trajectory 1 is 100 s and the period of trajectory 2 is 200 s. The average Δ*t* of tracking the sinusoidal trajectory with periods of 100 s is 4.972 s, and the average Δ*t* of tracking the sinusoidal trajectory with periods of 200 s is 5.056 s.

For the AUV used in this article, *T*_c_ is 0.166667 s and experiment data shows Δ*t* is about 5 s. Therefore, *N* is set as 30 in this paper.

## 5. Experimental Verification

**Figure 6:**
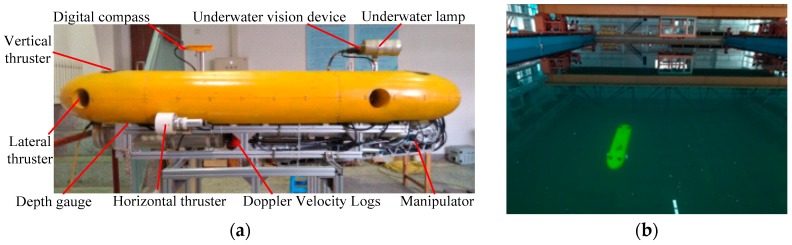
UVIC-I AUV and its autonomous operations in the pool. (**a**) UVIC-I prototype vehicle; and, (**b**) UVIC-I AUV during autonomous operations in the pool.

To evaluate the proposed methods on the UVIC-I AUV, the developed control algorithm was translated into the C/C++ programming language and then integrated within the vehicle control software. In this paper, the prediction horizon *p* is 80 and the control horizon *m* is 8 for the developed MPC algorithm. The UVIC-I AUV, as shown in Figure 6a, with a dimension of 2.0 m × 0.6 m × 0.54 m and a weigh of 205 Kg. It is a prototype vehicle that is designed for underwater autonomous operations. Experiments are obtained in a pool measuring 50 m × 30 m × 10 m, as shown in Figure 6b.

**Figure 7:**
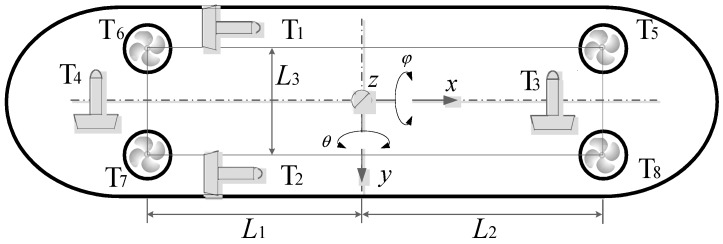
Thruster configuration of UVIC-I AUV.

UVIC-I AUV is equipped with eight thrusters, two to actuate surge, two to actuate sway and yaw, and four vertical thrusters to actuate heave, roll, and pitch, as shown in Figure 7, where *L*_1_ = 0.75 m, *L*_2_ = 0.85 m, *L*_3_ = 0.27 m [27]. A depth sensor, with an accuracy of 0.003 m, is installed on UVIC-I AUV for depth measurement, and the heave speed is obtained by depth sensor data differential. The depth and speed data are recorded on the UVIC-I AUV onboard memory for each control cycle. The data is read and analyzed offline.

The dynamic equation of UVIC-I in heave can be obtained with least square method [9,36], as follows:

(16) $$135.0727\ddot{z}+\left(214.9528-41.8985\left|\dot{z}\right|\right)\dot{z}=u$$

In our applications, UVIC-I AUV is designed for autonomous operations with a very low speed, and when it executes operations task, thrusters often reverse, resulting in AUV working nearby *v_z_* = 0 m/s. The vehicle moves with a max speed of 0.15 m/s, and when the vehicle executes operation missions, the speed is much lower than 0.15 m/s. The elements matrix A and matrix B, as obtained at *v_z_* = 0.15 m/s or −0.15 m/s, have less than 1% change in in comparison with at *v_z_* = 0.0 m/s. So, Equation (4) is linearized at *v_z_* = 0.0 m/s, and ***A***, ***B***, ***C*** can be represented as:

$$A=\left[\begin{array}{cc} 1 & 0.1464\\ 0 & 0.7670 \end{array}\right];\;B=\left[\begin{array}{c} 0.0001\\ 0.0011 \end{array}\right];\;C=\left[\begin{array}{cc} 1 & 0 \end{array}\right]$$

### 5.1. Experiment on MPC Method with a Real-Time Adjusting **Γ**_u_

#### 5.1.1. Experiments of Tracking Step Trajectory

For the MPC method with a real-time adjusting ***Γ**_u_* developed in this paper mainly focus on reducing settling time, experiments of tracking step trajectory is designed to verify the effectiveness. The following desired depth *z_d_*(*t*) in this paper is considered as:

(17) $$z_{d}\left(t\right)=\left\{ \begin{array}{ll} 1.0 & 0s\leq t<100s\\ 2.0 & 100s\leq t<200s\\ 1.0 & 200s\leq t<300s \end{array}\right.$$

**Figure 8:**
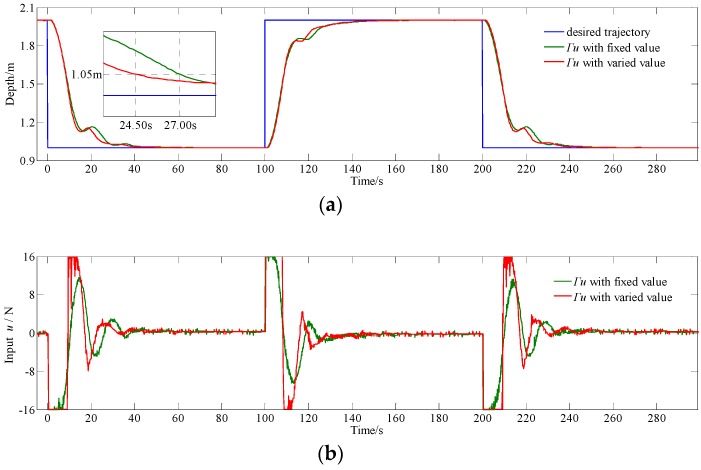
Contrast experiment result for model predictive control (MPC) with fixed ***Γ**_u_* and adjusting ***Γ**_u_* tracking step trajectory. (**a**) Depth data for MPC with fixed ***Γ**_u_* and adjusting ***Γ**_u_*; and, (**b**) input *u* for MPC with fixed ***Γ**_u_* and adjusting ***Γ**_u_*.

The experimental results based on MPC with fixed ***Γ**_u_* and adjusting ***Γ**_u_* are shown in Figure 8, and the time consumed for the error to be reduced from 1 m to 0.05 m is summarized in Table 1.

From data in Table 1, it can be seen that the settling time reduces from {27.00 s, 27.00 s 26.50 s} to {24.50 s, 26.17 s, 24.00 s}, with a decrease time of {2.50 s, 0.83 s, 2.50 s} separately, and an average settling time decrease 1.94 s. The experimental data indicates that MPC with a real-time adjusting ***Γ**_u_* can reduce the settling time compared with MPC with a fixed ***Γ**_u_* (***Γ**_u_* = ***I****_m_*_×*m*_).

Because of the current experimental conditions, experiments cannot be carried out at larger depths and depth variations. This is also an experimental content of the future work, and experiments need to be realized in the future lake test or the sea test.

When the error is large, the curve of input *u* in Figure 8b shows that MPC with real-time adjusting ***Γ**_u_* can change the input more rapidly than MPC with fixed ***Γ**_u_* to improve the response when the error is large. This is due to the fact that fixed ***Γ**_u_* set as ***I****_m_*_×*m*_ results in that the control increment vector is too conservative during the settling stage. When the error is small, both the MPC with varied ***Γ**_u_* and the MPC with fixed ***Γ**_u_* have a good depth keep characteristic. This is due to varied ***Γ**_u_* is set as ***I***_*m*×*m*_ by Equation (11), which causes the controller to perform conservatively to maintain the characteristics of reducing output disturbances and fluctuations on the steady stage.

#### 5.1.2. Experiments of Tracking other Trajectories Using MPC Method with a Real-Time Adjusting ***Γ**_u_*

**Figure 9:**
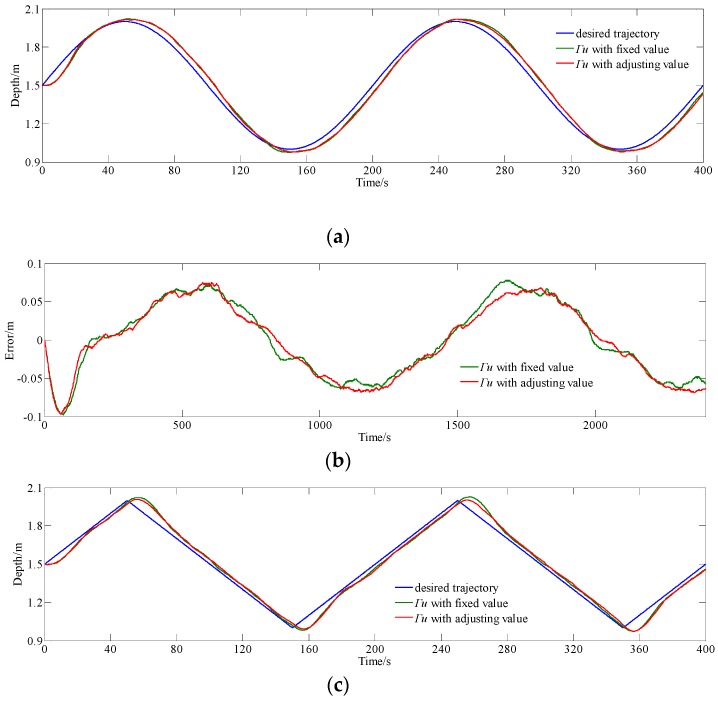

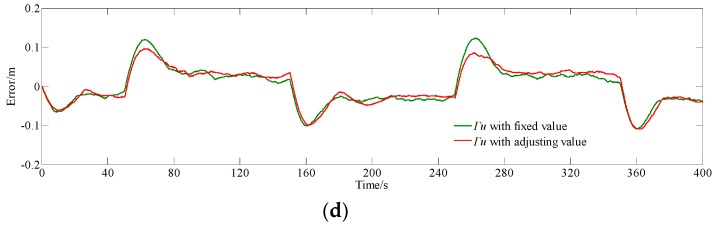
Contrast experiment result for MPC with fixed ***Γ**_u_* and adjusting ***Γ**_u_* tracking sinusoidal and triangular trajectories. (**a**) Depth data tracking sinusoidal trajectory; (**b**) error data tracking sinusoidal trajectory; (**c**) depth data tracking triangular trajectory; and, (**d**) error data tracking triangular trajectory.

In this section, experiments of tracking other trajectories using MPC method with a real-time adjusting ***Γ**_u_* are carried out to examine the control effect, as shown in Figure 9, and the results are summarized in Table 2. The following desired depth *z_d_*(*t*) is considered as:

A sinusoidal trajectory

(18) $$z_{d}=0.5\times sin\left(\pi t/100\right)+1.5$$

and a triangular trajectory

(19) $$z_{d}\left(t\right)=\left\{ \begin{array}{ll} 0.01t+1.5 & 0s\leq t<50s\\ -0.01\left(t-50\right)+2.0 & 50s\leq t<150s\\ 0.01\left(t-150\right)+1.0 & 150s\leq t<250s\\ -0.01\left(t-250\right)+2.0 & 250\leq t<350s\\ 0.01\left(t-350\right)+1.0 & 350\leq t<400s \end{array}\right.$$

From Figure 9b,d, it can be seen that the maximum of absolute error of adjusting ***Γ**_u_* MPC (with error intervals of [−0.097 m, 0.078 m] and [−0.109 m, 0.095 m]) is smaller than fixed ***Γ**_u_* MPC (with error intervals of [−0.098 m, 0.075 m] and [−0.109 m, 0.123 m]), and except for areas near the peak of absolute error, the error curves are similar to those that are based on MPC with fixed ***Γ**_u_* and adjusting ***Γ**_u_*.

From data in Table 2, it can be seen that average of absolute error and standard deviation of error changes from {0.04174 m, 0.04736 m} to {0.04067 m, 0.04677 m} when tracking sinusoidal trajectory, and from {0.04123 m, 0.04933 m} to {0.04154 m, 0.04934 m} when tracking triangular trajectory, using MPC with fixed ***Γ**_u_* and adjusting ***Γ**_u_*, respectively. The experimental results in Sections 5.1.1 and Sections 5.1.2 indicate that the MPC method with real-time adjusting ***Γ**_u_* can reduce settling time relatively on the premise of keeping the tracking accuracy.

### 5.2. Experiments on Simply Re-Planning the Desired Trajectory

#### 5.2.1. Experiments of Tracking Sinusoidal Trajectory

**Figure 10:**
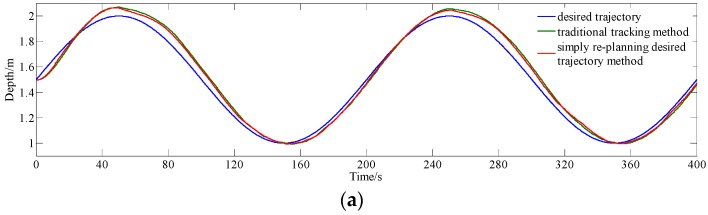

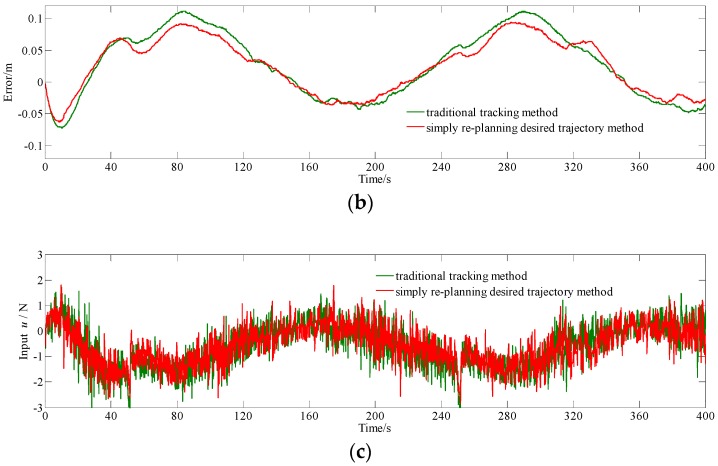
Comparative experiment results for simply re-planning the desired trajectory method and conventional tracking method with MPC tracking sinusoidal trajectory. (**a**) Depth data; (**b**) error data; and, (**c**) input *u.*

Experiments of the AUV tracking a trajectory of Equation (18) are carried out, and comparasive results for simply re-planning the desired trajectory method (i.e., treating the point *N* steps ahead on the desired trajectory as the present desired point method) and conventional tracking method (*N* = 0) are shown in Figure 10, and the results are summarized, as shown in Table 3.

From Figure 10a,b, in the early stage (i.e., 0–15 s), faster response and smaller error are obtained based on our new design, as compared with the conventional tracking method. From Figure 10a, it can be seen that the lag between the real trajectory and the desired one is also small based on the new design, in comparison with the result from the conventional MPC without trajectory re-planning. From Figure 10b, the maximum of absolute error from the proposed method (the error interval of [−0.063 m, 0.094 m]) is smaller than those from the conventional method (the error interval of [−0.072 m, 0.111 m]).

From data in Table 3, it can be seen that average of absolute error and standard deviation of error reduce from {0.04174 m, 0.04736 m} to {0.04067 m, 0.04677 m} when tracking sinusoidal trajectory, with decrease percentage of 14.80% and 17.15%, respectively, using simply re-planning the desired trajectory method and conventional tracking method. The experimental results indicate that the actual output trajectory with simply re-planning the desired trajectory method is closer to the desired trajectory than using conventional tracking method, which means that the lag between actual output trajectory and desired trajectory is reduced using simply re-planning the desired trajectory method.

#### 5.2.2. Experiments of Tracking Triangular Trajectory

**Figure 11:**
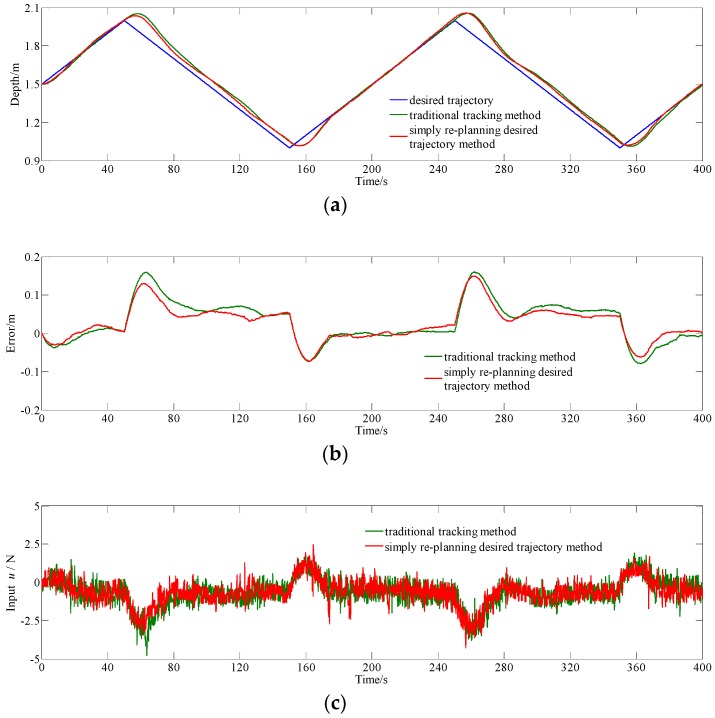
Comparative experiment results for simply re-planning the desired trajectory method and conventional tracking method with MPC tracking triangular trajectory. (**a**) depth data; (**b**) eError data; and (**c**) input *u.*

To validate simply re-planning desired trajectory method, experiments of tracking triangular trajectory are conducted. The desired depth *z_d_*(*t*), shown as Equation (19), is considered. Comparative results for simply re-planning the desired trajectory method and conventional tracking method are shown in Figure 11, and the results are summarized, as shown in Table 4.

From Figure 11a,b, at the beginning stage (i.e., 0–15 s), the improved MPC has a faster respond speed and smaller error by adding the re-planning loop. Similar to Figure 10, Figure 11a also shows that the proposed method can reduce the lag between the real trajectory and the desired one. From Figure 11b, in terms of the maximum of absolute error, the proposed method has better results with an error interval of [0.073 m, 0.150 m], when compared with the conventional method with an error interval of [−0.079 m, 0.161 m].

Data in Table 4 shows that simply re-planning desired trajectory method can reduce the average of absolute error from 0.04733 m to 0.03928 m, decreased by 17.01% and standard deviation of error from 0.05346 m to 0.04373 m, decreased by 18.20% as compared with conventional tracking method. Similar to the case of sinusoidal trajectory tracking, in the case of triangular trajectory tracking, the experimental results validate that the actual output trajectory with simply re-planning the desired trajectory method is closer to the desired trajectory than using conventional tracking method and the lag between actual output trajectory and desired trajectory is reduced using simply re-planning the desired trajectory method.

## 6. Conclusions

The contributions in this paper are on the application of improved MPC for AUVs. Real-time adjusting ***Γ**_u_*, according to the error method and simply re-planning the desired trajectory method are addressed. The proposed method of varying ***Γ**_u_* according the error online can improve MPC, mainly reflected by the reduction of settling time when tracking the step signal, and the method of re-planning the desired trajectory can optimize MPC by improving the tracking accuracy when tracking the continuous signal. Experimental results on UVIC-I AUV show that the improved MPC can reduce the settling time and a smaller lag is obtained by the developed re-planning desired trajectory in this paper. In this research, some issues are raised and the following work needs to be done in future. Firstly, we need to investigate how to on-line adjust the time interval *N* according to error to further improve the tracking precision. Secondly, simulation models need to be developed for AUV with six degrees of freedom and lake or sea test also need to be conducted at larger depths and depth variations to further validate the feasibility of the new design.

## Figures and Tables

**Table 1:** Settling Time Results of Figure 8.

	1st Settling Time	2nd Settling Time	3rd Settling Time	Average
Fixed ***Γ**_u_*	27.00 s	27.00 s	26.50 s	26.83 s
Adjusting ***Γ**_u_*	24.50 s	26.17 s	24.00 s	24.89 s
Decrease time	2.5 s	0.83 s	2.5 s	1.94 s

Note: The settling time in Table 1 is the time consumed from the controller receiving the desired point to the error reducing to 5% of the error.

**Table 2:** Statistical Result of Figure 9.

	Tracking Sinusoidal Trajectory		Tracking Triangular Trajectory	
	Average of Absolute Error	Standard Deviation of Error	Average of Absolute Error	Standard Deviation of Error
Fixed ***Γ**_u_*	0.04174 m	0.04736 m	0.04123 m	0.04933 m
Adjusting ***Γ**_u_*	0.04067 m	0.04677 m	0.04154 m	0.04934 m
Decrease percentage	2.56%	1.25%	−0.75%	−0.02%

**Table 3:** Statistical Result of Figure 10.

	Average of Absolute Error	Standard Deviation of Error
Conventional tracking method	0.05000 m	0.05196 m
Simply re-planning the desired trajectory method	0.04260 m	0.04305 m
Decrease percentage	14.80%	17.15%

**Table 4:** Statistical Results of Figure 11.

	Average of Absolute Error	Standard Deviation of Error
Conventional tracking method	0.04733 m	0.05346 m
Simply re-planning the desired trajectory method	0.03928 m	0.04373 m
Decrease percentage	17.01%	18.20%
